# Traditional bone setter practices and the interaction with biomedical care in the treatment of hip fractures in The Gambia: A qualitative study

**DOI:** 10.1371/journal.pgph.0006582

**Published:** 2026-07-14

**Authors:** Awa Touray, Kimberly Lakin, Rachael Gooberman-Hill, Omar Cessay, Lucy Gates, Jainaba Badjie, Tida Saidy, Kaddy Darboe, Matthew L. Costa, Celia L. Gregson, Kate A. Ward, Kebba Marenah, Sarah Drew

**Affiliations:** 1 Faculty of Environmental and Life Sciences, School of Health Sciences, University of Southampton, Southampton, United Kingdom; 2 MRC Unit the Gambia at LSHTM, Fajara, The Gambia; 3 Global Health and Ageing Research Unit, Bristol Medical School, University of Bristol, Bristol, United Kingdom; 4 School of Healthcare Enterprise and Innovation, Faculty of Medicine, University of Southampton, Southampton, United Kingdom; 5 Oxford Trauma and Emergency Care, Nuffield Department of Orthopaedics, Rheumatology and Musculoskeletal Sciences, University of Oxford, Oxford, United Kingdom; 6 The Health Research Unit Zimbabwe, Biomedical Research and Training Institute, Harare, Zimbabwe; 7 MRC Lifecourse Epidemiology, Human Development and Health, University of Southampton, Southampton, United Kingdom; 8 Department of Orthopaedics and Trauma, Edward Francis Small Teaching Hospital, Banjul, The Gambia; University of Global Health Equity, RWANDA

## Abstract

A growing ageing population in Africa underscores the need to reorient health systems to provide care for non-communicable conditions impacting older populations such as hip fractures. In The Gambia, the number of hip fractures are expected to quadruple for both men and women in the next 30 years. This is comparable to hip fracture incidence rates in other South and West African countries. Moreover, mortality rates post-hip fracture are 2–3 fold higher in populations from the African Region than for those in other high-income countries. Globally, in many countries including in The Gambia, musculoskeletal conditions are treated in a pluralistic medical system by both biomedical healthcare professionals and traditional bone setters (TBS). Further research is required to understand the interactions between these different therapeutic modalities in the treatment of fractures, including hip fractures – a life changing injury. Such evidence is important for developing collaborative initiatives between traditional and biomedical practitioners. This study sought to describe the practices and procedures used by TBS to treat hip fractures and their interactions between with biomedical care in The Gambia. In-depth interviews were conducted with 16 TBS and 38 healthcare professionals involved in hip fracture care provision in both rural and urban regions of the country. Data were analysed using an inductive thematic analysis. TBS used different modalities to identify a hip fracture. Most TBS misidentified and treated hip fractures as dislocations. On account of this, treatment consisted of pulling, massaging, and kneading the limb back into the hip socket. During this process, TBS applied concoctions of herbs and butters followed by splinting and tying the limb. Few TBS provided pain relief medication during treatment and most advised an extensive period of immobilisation of the limb. Follow up visits reviewed recovery. The narratives of both TBS and healthcare professionals further highlighted the informal referral of hip fracture patients from TBS to biomedical care to receive radiographs, analgesia, and for other ailments. While some biomedical healthcare professionals maintained that TBS should have no role in hip fracture care, others noted the importance of regulation of TBS practices and training initiatives. Considering these findings, we propose short-, medium- and long-term recommendations for the integration of traditional and biomedical sectors in the treatment of hip fractures in The Gambia. This includes the need to establish regulatory mechanisms for TBS practices and patient referral pathways between biomedical and TBS care.

## Introduction

Reflecting the trend of other African nations, The Gambia is experiencing rapid demographic change. Life expectancy has increased and, from 2013 to 2024, the number of people aged 65 years and older grew by 25.8% [1]. A growing ageing population comes with the inevitable increase in fragility fractures, including hip fractures [2]. In The Gambia, our recent work estimated hip fracture incidence rates, for those age ≥ 40 years, to be 51.7 for women and 28.1 for men per 100,000 [2]. These rates were broadly comparable to other West African settings including, Zimbabwe, Botswana, and for Black South Africans [3,4]. In Zimbabwe, the number of hip fractures are expected to increase more than 2.5-fold in the coming decades whereas, in The Gambia, hip fracture cases will almost quadruple [3,4].

Therefore, in these contexts, health systems which have hitherto centred on the delivery of communicable disease care, must now reorient to provide orthopaedic trauma care, often to patients with co-morbid, non-communicable diseases. Fracture care clinical guidelines by the African Orthopaedic (AO) Alliance specify the need for emergency trauma assessment, effective pain management, diagnosis through radiographic imaging with 24 hours, operative management for fracture fixation, weight bearing post-surgery, and post fracture rehabilitation [2,5]. Yet, in The Gambia, our work identified that approximately 7% of current inpatient care is dedicated to orthopaedics, with less than one orthopaedic surgeon available for each 100,000 adults. Therefore, in many parts of Africa, including in The Gambia, fractures are treated ‘pluralistically’ by both traditional and biomedical practitioners. While this may be partly due to the under provision of health services and the high costs of biomedical healthcare, the pluralistic treatment of fractures is also driven by cultural beliefs and the longstanding patronage held by traditional medical practitioners [2,6,7].

The notion of medical pluralism has been conceptualised in different ways. As Krause et al. [8] explain, early conceptualisations of “medical pluralism” referred to the idea of distinct but parallel medical sectors which patients use sequentially or at the same time. In addition to ‘formal’ biomedical care, this includes the use of traditional medicine. Traditional medical care has a long-standing tradition in many societies, such as in India and China, and comprise of the “practices, skills, knowledge and philosophies... which are distinct from and pre-date biomedicine” [9]. Recent debates within medical anthropology have called for a shift away from understanding medical pluralism as consisting of separate, distinct sectors and towards considering the “mixture and intersections of different therapeutic practices” [8,10]. Within this conceptualisation, traditional and biomedical sectors do not have defined boundaries. Rather, their boundaries are constantly reconfigured in response to interactions between the sectors [8]. Kleinman [11]’s model of healthcare systems, for instance, has articulated the interrelations between these different therapeutic traditions. Within this model, patients seek care from three overlapping sectors: the ‘popular’ or lay sector (self-care and home remedies), the ‘professional’ or biomedical care (encompassing the biological and physiological sciences and clinical medicine), and the ‘folk’ sector (traditional medical practitioners) [11,12].

While interactions between biomedical and traditional care can occur when patients seek care from these sectors at various stages of treatment, it may also be evident through formal or informal collaborative activities between biomedical and traditional healthcare professionals [13]. Empirical work has shed light on this interaction by examining the treatment of various conditions, such as mental illnesses and hypertension [13,14]. Studies in the Philippines, for instance, have shown that patients may choose to seek care from different therapeutic modalities depending on a confluence of factors such as, understandings of their illness, social relationships, and their expectations of care [15]. However, Pham et al.’s [13] work in Nepal described how biomedical healthcare professionals enabled patients to see a traditional healer, alongside their medical treatment, to receive blessings and prevent supernatural involvement. To better respond to patients’ expectations, the World Health Organization (WHO) called for the integration of “traditional, complementary, and integrative medicine” (TCIM) within their recently published Traditional Medicine Strategy (2025–2034) [16]. A key objective of this strategy is to reorient national health systems to integrate safe and effective TCIM, while instituting regulatory mechanisms, training programmes, and policy frameworks.

In many regions of the world, including in Africa, South America, and India, Traditional Bone Setters (TBS) are an important form of traditional medicine and experience widespread patronage for the treatment of musculoskeletal injuries and fractures [17]. TBS are a type of traditional healer with specialisation in treating musculoskeletal injuries within the community [18]. Empirical work has explored bone setting practices in Nigeria, Ghana, and Tanzania [19–21]. These studies have outlined a largely standard procedure of treatment through direct manipulation, splinting with materials such as bamboo and palm sticks, bandaging, and application of herbal creams [6,18,22]. The use of analgesics during treatment however varies, with some TBS providing herbs, alcohol, and over-the-counter pain medication, while others offering none. Similarly, though these studies suggest that the majority of TBS solely diagnose fractures through physical assessment, a small number of studies [21,23] have identified that some TBS rely on X-rays performed at hospitals. To promote healing and to address beliefs around the causes of the injury, TBS may use incantations during the treatment process [24]. While TBS treatment methods may be affective for closed fractures treatment of more complex injuries, including hip fractures, may lead to significant complications including loss of limbs due to gangrene, osteomyelitis (infection of the bone), and lifelong disabilities from non-union and malunion [22].

Our work [2] recently assessed fracture service availability and readiness provided by both biomedical and traditional health practitioners in The Gambia. The assessment revealed that 60% of patients with hip fractures, presenting to one of the 150 facilities surveyed, had already engaged with a TBS; however, little is known about the practices of TBS or how they interact with biomedical care in the Gambia. Some studies have examined the overlapping treatment practices between biomedical care and TBS in other countries in Africa. For instance, work from Chad [18] has shown how local bonesetters, or “Jabari”, worked with physicians to provide trauma care in the community. However, Krah et al.’s [20] study in Ghana identified persistent challenges to integrating traditional medicine and biomedical care practices, including healthcare professionals’ limited understanding of traditional healers’ knowledge and practices. Kleinman [11] explains that interactions between the different therapeutic sectors of local health system occur primarily because patients pass between them in response to their “illness trajectories”. Our recent work in The Gambia [25] identified complex hip fracture care pathways with patients navigating between TBS, public, and private health services at different stages of the treatment journey. Various intersecting factors shaped these treatment journeys including, dependency on caregivers, organisational barriers in hospitals such as long wait times, and fear of biomedical care.

Therefore, and as outlined in the WHO’s Traditional Medicine Strategy [16], there is a need to not only understand TBS treatment practices, but to also explore how and to what extent TBS interact with biomedical care. Such evidence is vital for the development of evidence-based, context-specific strategies and initiatives to better integrate biomedical and traditional care. In this study, we aimed to describe the practices and procedures involved in the treatment of hip fractures by TBS in The Gambia. We focused on the examination of the interaction (if any) between biomedical and traditional sectors in hip fracture care provision. Within this study, we define biomedicine as the science-based model of medicine that conceptualises health and illness primarily through biological, chemical, and physical mechanisms [11,12]. Drawing on these insights, we propose recommendations for policymakers and practitioners to consider for integrating traditional and biomedical care within the national healthcare system.

## Methods

### Study context

The Gambia is a low-income country located in West Africa with a population of 2.7 million people, 35% of whom reside in rural areas [26]. Public health care is delivered through a three-tiered health system consisting of primary-, secondary-, and tertiary-level facilities. At the central level, the Ministry of Health is responsible for setting national health priorities and regulations. Primary health care is delivered through village health workers based in village health facilities. However, in recent years, primary health care provision has severely deteriorated, with an average of 40% having access across The Gambia [27]. Health care is therefore preferentially sought at secondary-level health centres and tertiary-level District, General, and teaching hospitals.

At the time of the study, from 2022 to 2025, most public orthopaedic care in the country was provided in one central hospital – the Edward Francis Small Teaching Hospital – which is the only public tertiary-level facility in The Gambia. Between June 2022 and June 2023, 78 patients over the age of 40 presented to Edward Francis Small Teaching Hospital with hip fractures, with 43 (59%) treated surgically. Two other private clinics and a non-governmental health facility also had capacity to treat hip fractures surgically. Our [2] assessment carried out in rural and urban regions of The Gambia found that only a third of tertiary-level facilities, including private hospitals, had access to a physiotherapist; few other facility types had physiotherapy access. Our study [2] also collected data from 42 TBS across The Gambia; however, this is likely an underestimation due to the absence of a formal register of TBS. Though the processes of bone setting vary between TBS, treatment typically follows six stages: 1) fracture identification, 2) palpation and massaging of the injury, 3) application of shea butter and herbs (sometimes together with recitation of incantations), 4) splinting and tying, and 5) immobilisation. Processes of treatment are discussed in detail below.

### Study design

This study was conducted as part of the “Fractures in sub-Saharan Africa: Epidemiology, Economic impact and Ethnography” study (the Fractures-E3 study) [2,3]. The protocol for this study has been published [28]. The study draws on data collected from semi-structured, in-depth interviews with TBS and healthcare professionals across the care pathway who deliver hip fracture care in The Gambia. Interviews were complemented by observations of TBS practices to understand treatment settings and procedures for fracture care.

### Participant recruitment

Identification and recruitment of TBS occurred between May 2022 and September 2024. Potential TBS participants were identified as part of recruitment for other work packages in the Fractures-E3 study. Previously recruited TBS who had agreed to further contact were approached in person by the study team in their homes or other places of business. TBS, experienced with treating fractures, and practicing in the Greater Banjul (urban) and West Kiang (rural) areas, were eligible to participate. A total of 19 TBS were approached of whom 16 consented to participate: 10 from Kanifing Municipality, West Coast region, and 6 from West Kiang. Observations of treatment settings and practices of five TBS were conducted.

In addition, healthcare professionals were recruited from both public and private health in Banjul**,** Kanifing Municipality and West Coast Region, facilities including a Tertiary Referral Hospital (the Edward Francis Small Teaching Hospital), a District Hospital), a Medical Research Centre, and two Private Clinics. This was to enable the inclusion of participants from a range of facilities involved in various aspects of hip fracture care, including tertiary centres with capacity to provide operative care and lower-level facilities involved in the referral of hip fracture patients to higher-level care. Purposive sampling was used to recruit healthcare professionals involved in hip fracture management across the care pathway, including medical officers, orthopaedic surgeons, nurses, and physiotherapists. The study team contacted potential participants by phone to provide study details, before arranging an interview. A total of 38 healthcare professionals consented to participate, 35 from Greater Banjul and the West Coast, and 3 from West Kiang. Determining sample size in qualitative research remains a contentious issue and researchers have since called for a shift away from assessing the numerical input of study participants towards determining the contribution of new knowledge from the analysis [29]. Final sample size for both TBS and healthcare professionals was guided by the concept of information power, in which the study’s sample size was determined as the data progressed by assessing the data collected against the achievement of the research aims [29]. This assessment was carried out at stages throughout the process of data collection, with recruitment of study participants ceasing when sufficient data had been collected to achieve information power.

### Data collection

Interviews with TBS were conducted in their homes or other places of business and interviews with healthcare professionals were conducted within healthcare facilities. Interview were conducted by Gambian-based researchers (OC, KD, TS, and AT) in Mandinka or Wolof languages, as requested by the TBS and healthcare professionals. The interviews were structured using a topic guide with questions exploring TBS practices relating specifically to hip fracture care, acquisition of bone setting knowledge, and their interactions and views of biomedical care (S1 Text). However, at times, TBS did describe the provision of fracture care more generally. The topic guide was developed by the study team with a diverse range of expertise, including social scientists, Gambian researchers and healthcare professionals involved in the delivery of hip fracture care, including orthopaedic surgeons and physicians (including those working within the Gambia). Each member of the team thus provided complimentary expertise on the socio-cultural context of The Gambia and the nature of hip fracture care provision which informed data analysis. Interviews and observations were carried out by Gambian researchers. Their positionality conferred multiple advantages, including linguistic fluency, cultural competence and in-depth contextual insights. Gambian researchers were well positioned to build rapport with participants, thereby improving data richness. Interviews lasted 45–75 minutes and, with consent, were audio recorded. Observations conducted in TBS homes or other places of business provided further insight on the procedures involved in the treatment of hip fractures, materials used, as well as the setting within which treatment was provided. Observation checklists were developed to guide data collection and observations were recorded in the form of fieldnotes (S3 Text). A total of eight hours of observation of TBS practice was conducted. Interviews with healthcare professionals were conducted in the healthcare facilities in their place of work, by three researchers (OC, KD, and TS). Interviews explored experiences and views of TBS practices and their current interactions with TBS (if any). This data was collected as part of wider discussions about factors that impact on the delivery of hip fracture care in The Gambia (S2 Text). The interviews were conducted in Mandinka, Wolof, or English, depending on participants’ preferences.

### Data analysis

Data generated from the interviews and observations were analysed thematically, using Braun and Clarke’s inductive thematic analysis approach [30]. The process of analysis occurred concurrently with data collection and began with the recording of analytical memos or short reflective notes by members of the study team in fieldwork diaries. The study team comprised social scientists, Gambian researchers, healthcare professionals (including those working in The Gambia), orthopaedic surgeons and physicians. As such, the team brought complimentary perspectives to understanding the phenomena. For instance, social scientists viewed TBS practices through the lens of medical pluralism and associated theory. Gambian researchers provided critical insights into their integral role within society and the healthcare system. Reflexive practices were embedded throughout the research process to scrutinise how these different perspectives shaped data interpretation. This was achieved through the use of analytical memos/ reflective note-taking, with insights shared during collaborative analytic meetings. This process supported a balanced and more nuanced understanding of TBS practices [31]. Audio recordings of interviews conducted in Mandinka and Wolof were translated into English by Gambian-based researchers with understanding of local context; once translated interviews were then transcribed verbatim, during which transcripts were anonymised. Full fieldnotes were developed from observations of TBS treatment settings and practices. Interview transcripts and observational fieldnotes were imported into NVivo qualitative analysis software which facilitated thematic analysis.

We followed Braun and Clarke’s [30] six step process of 1) data familiarisation, 2) generation of initial codes, 3) identification of themes, 4) reviewing of themes, 5) defining themes, and 6) report write-up. Transcripts and fieldnotes were read to develop a preliminary understanding of the data and initial codes and themes were then generated. Coding was conducted independently by two members of the study team (AT and KL). The researchers then met to discuss the coded data, as well as to compare and contrast the codes generated. During these collaborative discussions, AT and KL revisited the data, examined areas of agreement and divergence, and worked together to resolve any discrepancies. For instance, in distinguishing between the various procedures TBS followed in treating hip fractures. This process supported the refinement of the coding framework and enhanced the overall validity and rigour of the analysis [30]. The broader research team held regular collaborative meetings to review themes and sub-themes. Additionally, the contextual expertise of Gambian researchers ensured that the interpretation of data was credible and grounded in the local context [31]. As part of these meetings, researchers reflected on how their professional and personal backgrounds may influence the interpretation of data. These collaborative discussions thus extended the analysis by illuminating the ways in which researchers’ assumptions and values shaped their understanding of study findings. For instance, this was important when it came to faithfully reporting the various ways that TBS acquired bone setting knowledge. Collaborative meetings also enable the research team to resolve any discrepancies in the themes/sub-themes generated and contributed towards further refinement.

### Research ethics

Ethical approval was provided by The MRC The Gambian Scientific Coordinating Committee (SCC 22975). The Ministry of Health Services and Gambian Government provided approval for the research in public healthcare facilities. Healthcare professionals and TBS were required to sign, mark or thumbprint (with a witness) a consent form prior to data collection. This provided the study researchers with permission to shadow them in their practice or place of work. Consent forms were in English (the standard written language in The Gambia), with content explained in Mandinka or Wolof as needed. In addition, TBS provided additional consent for the researcher to take photographs of their practices. Study participants were reimbursed for any transportation-related costs. They were also provided with a “token of appreciation” to thank them for taking part in the study. This is in keeping with approaches that recognise the important contribution made by participants to research that takes their time.

## Results

Of the 16 TBS recruited, only one was female (Table 1). 6 TBS practiced in rural West Kiang, and 10 in urban Greater Banjul and the West Coast. Within the 38 healthcare professionals recruited, 13 professional groups were represented, including orthopaedic surgeons, medical officers and physiotherapists (Table 2). Most [n = 26 (68.4%)] healthcare professionals were male, only one was an orthopaedic surgeon.

**Table 1:** Characteristics of traditional bone setters recruited in the study.

Traditional Bone Setter Characteristics		Total n = 16 n (%)
Sex	Male	15(93.8)
	Female	1(6.2)
Location	Rural	6(37.5)
	Urban	10(62.5)
Years of practice	≤10 years	0(0)
	11-15 years	2(12.5)
	16-20 years	4(25.0)
	>20 years	8(50.0)
	Not reported	2(12.5)

**Table 2:** Characteristics of healthcare professionals involved in hip fracture in The Gambia.

Healthcare Professional Characteristics		Total n = 38 n (%)
Sex	Male	26(68.4)
	Female	12(31.6)
Role	Administrators	5 (13.2)
	Anesthetist Nurse	4(10.5)
	General Nurse	5(13.2)
	Pre-operation Nurse	2 (5.2)
	Recovery Nurse	2(5.2)
	Theatre Nurse	3 (7.9)
	Triage Nurse	3(7.9)
	Matron	1(2.6)
	Medical Officer	5(13.2)
	Orthopedic Surgeon	1(2.6)
	Physiotherapist	2(5.2)
	Resident Doctors	3(7.9)
	X-ray Technicians	2(5.2)
Recruitment site	Urban private clinics	9(23.7)
	Peri-urban District Hospital	1(2.6)
	Urban Tertiary Hospital	21(55.3)
	Rural Medical Research Centre	3(7.9)
	Rural General Hospital	4(10.5)

**Fig 1:**
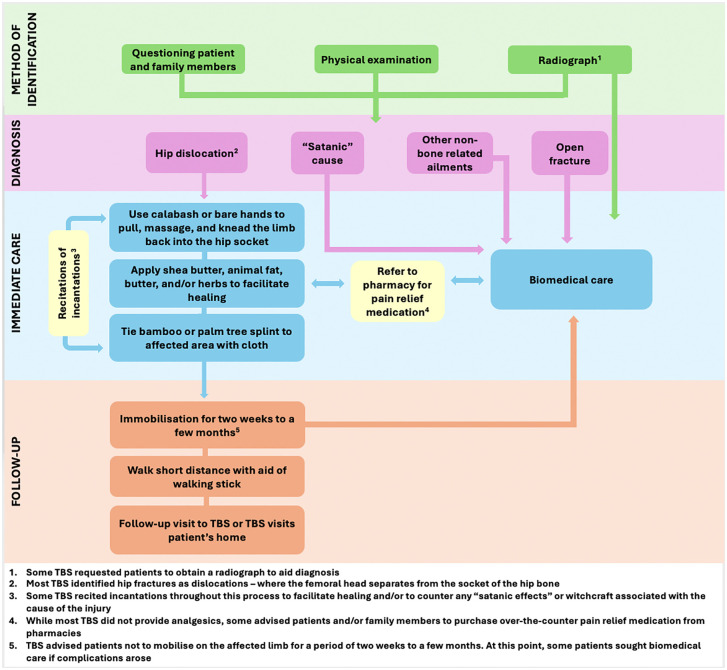
Practices and procedures of TBS in the treatment of hip fractures in The Gambia.

Below, we present findings from the analysis of interviews with TBS. These comprise two overarching themes, the first of which outlines “TBS characteristics and treatment settings” and the second of which characterises “TBS practices and procedures in treating hip fractures”. Within the latter, three sub-themes describe the identification, treatment, and post-treatment practices of TBS. Findings from the analysis of interviews with both healthcare professionals and TBS are shown through themes that explore the “Interaction between biomedical and traditional sectors in the treatment of hip fractures” and “Recommendations from TBS and biomedical healthcare professionals”. Sub-themes analyse TBS perceptions of biomedical care, healthcare professionals’ views of TBS practices, and the existing interactions between these care sectors (Fig 1).

### TBS characteristics and treatment settings

Most TBS interviewed learned the practice of bone setting at a very young age. Their knowledge of bone setting was acquired initially through observation of their teachers’ skills – typically a family member – before they were gradually given responsibility to manage bone-related injuries under guidance. Once they were proficient, TBS were able to practice independently.

*“...five years [old] you start learning about setting bone, […] you are seeing everything they do until the time they ask you to manage a sprain and then finally to heal broken bones. You will be taught everything you need to know till [...] you can practice alone outside the family.” (* ***TBS 02, Male).***

However, this was not always the case. Some TBS mentioned not receiving any training in bone setting and that their knowledge of treating bone-related injuries was a gift from God.

Most TBS emphasised that bone setting knowledge is strictly passed down the male family line as had been the case for generations. Yet, some mentioned training their daughters in bone setting and indeed the only female TBS we interviewed had been trained by her grandmother. Some TBS narrated an ‘origin story’ of how their forebearers acquired the knowledge of bone setting. While versions of this story varied between the TBS, it largely centred around their forefather’s gaining the skills in bone setting after treating an injured lion. Almost all TBS interviewed indicated that they did not keep any record of their patients and no documentation of bone setting knowledge and practices.

For most TBS, bone setting was not their primary occupation or source of income. They worked as teachers, builders, framers, electricians, and business owners, which provided a steady income for their family. As one TBS described, the income from solely practicing as a TBS was insufficient, partly as they often did not charge patients a standard fee. Instead, TBS asked patients to pay what they could afford, which was sometimes a small amount. TBS felt they were “blessed” by practicing bone setting and that these blessings were worth more than money. We expand on this below.

*“The reason why I have added this other [job] too is that I cannot only depend all my living on this bone setting as a responsible family man because [...] if you tell them your treatment fee some will tell you, “I don’t have that amount” and you will not have any option rather than to assist them.”* ***(TBS 05, Male).***

Given the diversity of their primary occupations, TBS treated patients in a variety of settings. While most TBS practiced bone setting in their homes, others attended to patients in their places of work. Moreover, most TBS described visiting patients’ homes to treat significant fractures, such as hip fractures, and especially if the patient was an older adult or from a rural area.

*“… mostly when you have hip fracture we use to treat you at your home and we fix it there because if you enter into a car it does not favour you due to the bad road conditions. So that is why we go to your home and treat your there and will be visiting you from time to time you till you are fully recovered.”* ***(TBS 08, Male).***

Overall, the TBS interviews indicate variation in the acquisition of bone setting knowledge. Though the occupation was seen as patriarchal by some TBS, others were open to training their daughters whilst some claimed to have received no training at all.

### TBS practices and procedures in treating hip fractures

#### Identification of a hip fracture.

When asked to describe the procedure of treating hip fractures, many TBS spoke about the need to first establish a rapport with their patient to empathise with their pain and foster trust.

*“When someone comes to me with a broken bone, I will welcome and help the person to feel relief because they are in pain. I will share jokes with them until the person feels comfortable, and even if I am not able to treat them”* ***(TBS 016, female).***

Examination of the injury would begin with the TBS questioning the patient and/or their family to ascertain the cause and extent of the injury. TBS reported that hip fractures could have various causes including, nutritional deficiencies, as well as “satanic causes” or “witchcraft”. As we describe below, some TBS mentioned that they would not treat a patient if a “satanic cause” was suspected as some believed that, by doing so, they too might also be affected by witchcraft. Nevertheless, many agreed to treating most hip fractures in older adults and, in these cases, they visited patients in their home.

*“I do not just visit people like that [those affected by witchcraft], but if I realise that if this individual is an elderly person and considering his condition, I can trust Allah [God] and know that he is one and he will support me. Then I will visit the patient in his house and treat.”* ***(TBS 04, male).***

After questioning the patient, the TBS would begin a physical examination which mostly involved palpating the affected area to confirm the type of injury. During this process, some TBS compared the length of patients’ legs to one another. TBS explained that, in hip fractures, they would observe that the affected leg would be shorter and facing outwards. Some described identifying a hip fracture purely through their intuition. Other TBS, from both rural and urban areas, requested radiographs to aid identification and encouraged patients to obtain a radiograph before visiting them:

*“Many will do X-ray first before calling me and when others call me without X-ray I will tell them to go for an X-ray first…. Many a times if they did an X-ray that’s what makes the work easy for me because when I check the X-ray I can tell whether it is dislocated or broken.” (* ***TBS 03, male).***

However, not all TBS used radiographs solely for diagnosis. Some requested patients to obtain an X-ray to check the healing progress and this, in turn, enabled patients to trust in the efficacy of their treatment:

*“[I will tell them to get an x-ray] if I want my patients to know the before and after of receiving treatment. If not I will be the only one to know how well I have healed them “* ***(TBS 01, male).***

In their descriptions of fractures, most TBS described hip fractures as dislocations – where the femoral head separates from the socket of the hip bone. For instance:

*“So, the hip has a hole, there is a hole there, the join at the last end has a hole you understand, the hole has a small stick like and when person falls or has an accident it that is the place that detached.”* ***(TBS 03, male).***

#### Process of treating a fracture.

**Fig 2:**
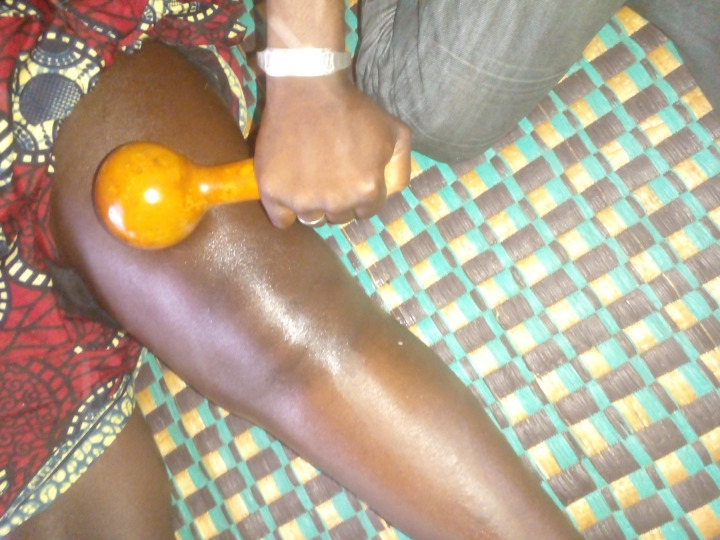
A TBS uses a heated calabash to massage a patient’s affected limb.

Most TBS subsequently treated hip fractures as dislocations which involved pulling, massaging, and kneading the limb back into the hip socket. Some TBS used their bare hands while others used a heated calabash (gourd fruit) to massage the affected area (see Fig 2). TBS described listening for a sound to indicate alignment:

*“[...] then you press it and the bone goes back in to that hole, you will hear a sound “cok”, the moment you heard the sound “cok” know that it is back to its place [...] When I am done then I will apply shea butter and other creams I have here that I bought from the markets and shops.”* ***(TBS 03, male).***

**Fig 3:**
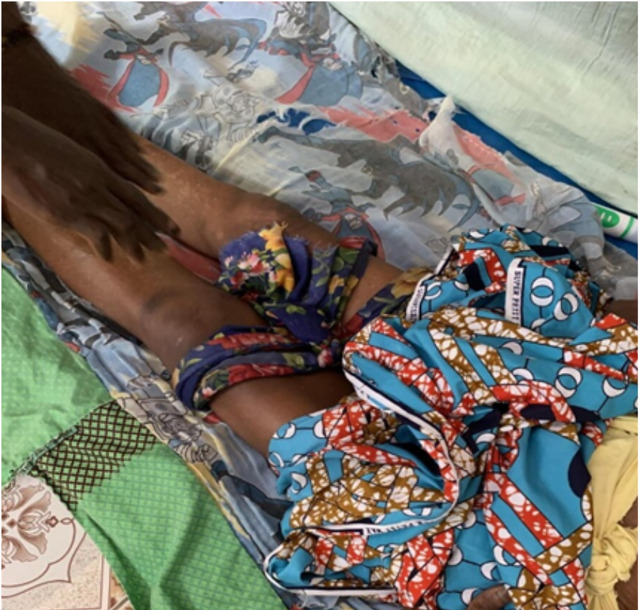
The affected hip and limb of a patient is tied using old cloths.

**Fig 4:**
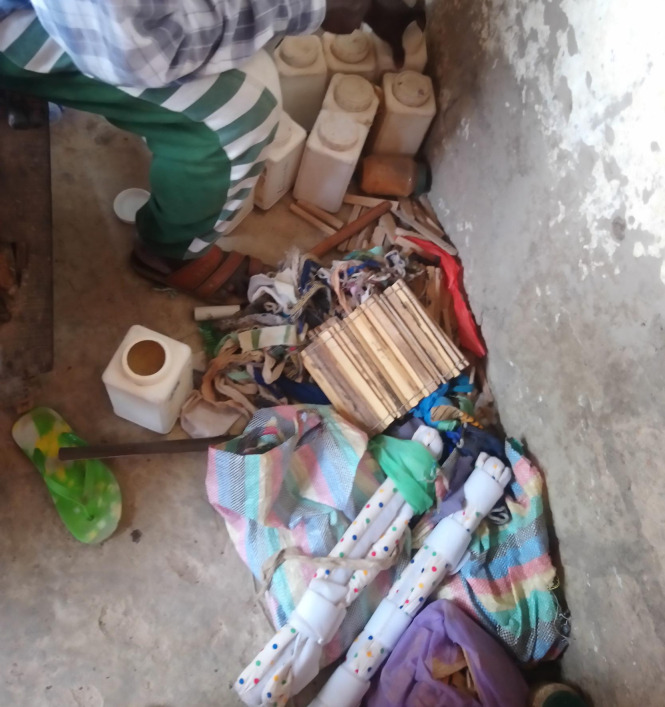
The treatment materials used by a TBS including, shea butter, herbs, old cloth, and a wooden splint.

**Fig 5:**
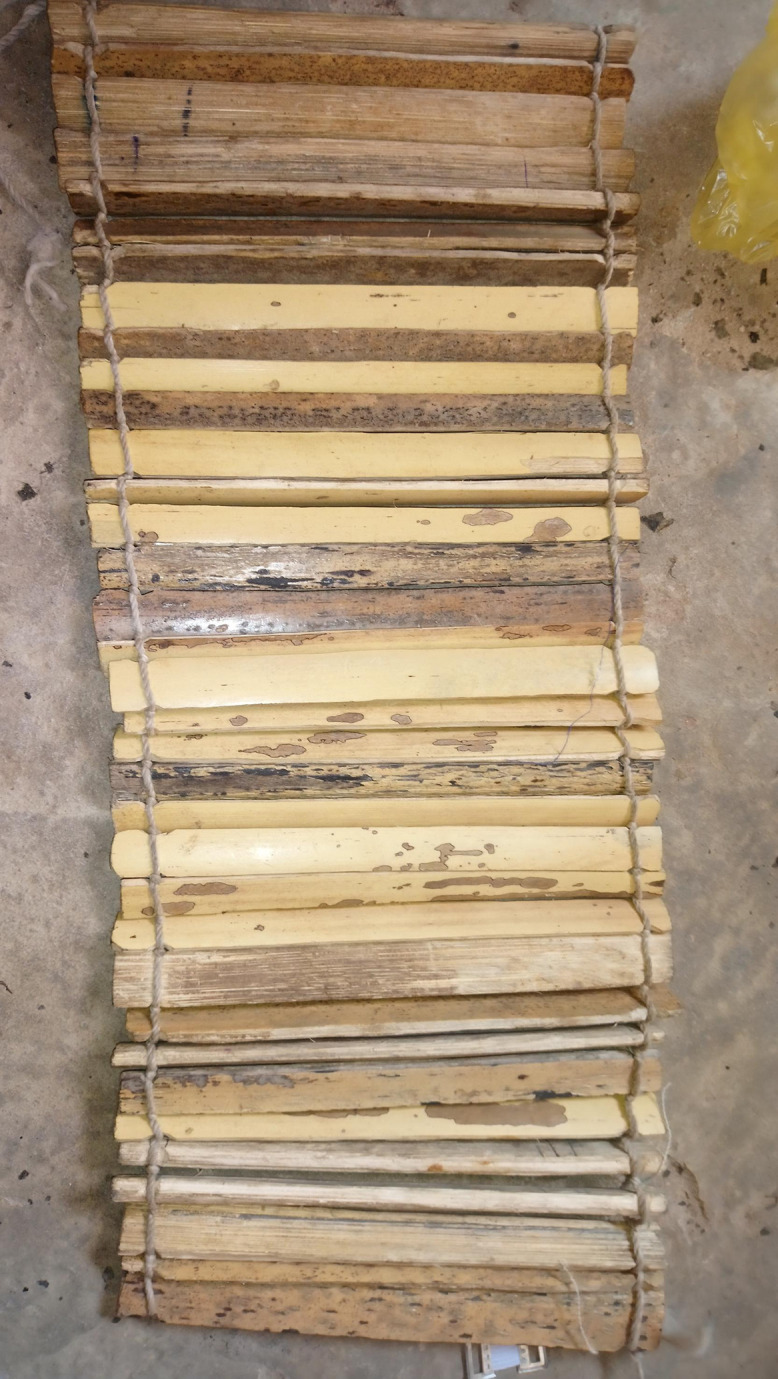
Wooden splint used for hip fractures.

As this TBS mentions, shea butter, animal fat, butter, and/or herbs are applied to the hip to facilitate healing which is then tied using a cloth (see Figs 3 and 4). During the observations, and as shown in Fig 5, TBS demonstrated how a splint is created out of bamboo or palm tree and tied to the affected area to prevent movement.

*“I use cloths to wrap, I buy second-hand cotton bed sheet, after aligning the bones with incantations I used sticks in a manner that the bones won’t be able to move before holding each other [...] you see that bamboo tree that is what we cut into pieces and aligned them on the affected part and tie.”* ***(TBS 02, male).***

Throughout this process, some TBS mentioned reciting incantations, usually verses from the Quran, to facilitate healing. The incantations also served to counter any “satanic effects” or witchcraft associated with the cause of the injury. Some TBS followed other practices, such as burying the cloth used to tie the fracture to prevent others from being injured in the same way.

“...*.one of the things I am concerned about is the piece of cloth, I ask them to bring back the piece of cloth when they recover fully. If you throw the piece of cloth, when someone’s body is not clean [spiritually], or prone to many things, the person might hit something, fall and break a bone, yes, it [touching the cloth] can cause that. But if the person is far and will not be able to bring it back here [to the bone setter], I advise them to dig a hole and put the piece of cloth inside the hole and bury it with sand to prevent another person from becoming a victim”* ***(TBS 014, male).***

TBS acknowledged the procedure of treating hip fractures caused considerable pain for the patient. Some TBS used herbal mixtures, together with incantations to try and help relieve the pain. However, most TBS did not provide methods intended to directly relieve or reduce pain. Others advised patients and/or family members to purchase over-the-counter pain relief medication from pharmacies.

#### Immobilisation and follow-up.

**Fig 6:**
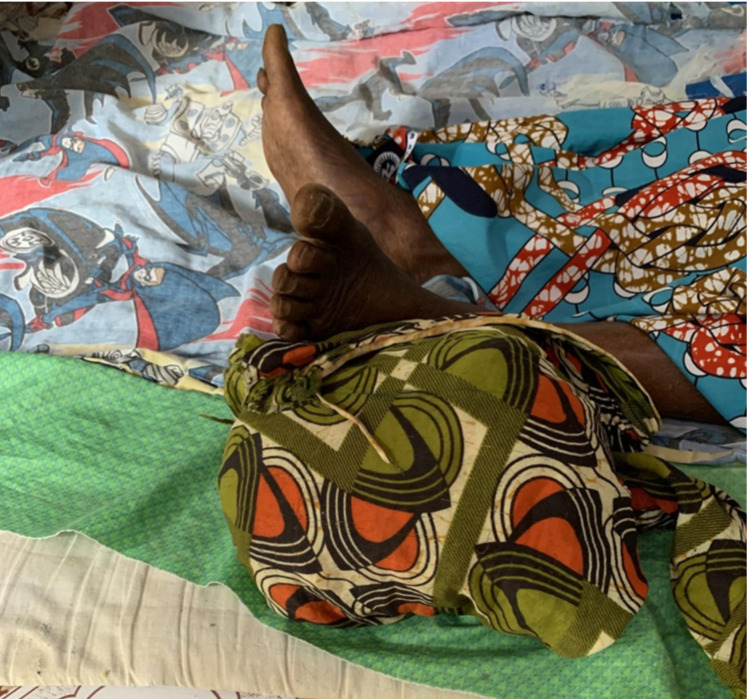
A patient’s leg is immobilised using a cement block wrapped in cloth.

After treatment, TBS advised patients to remain immobile until they deemed the fracture healed. This ranged from a period of two weeks to a few months. As shown in Fig 6, patients were advised to stay on the floor, and their limb was typically immobilised by placing cement blocks on either side of the limb to prevent movement (internal rotation of the limb towards the body or external rotation of the limb away from the body). TBS determined if the fracture was healed during subsequent follow up visits. The time for recovery varied anywhere from two weeks up to four months, but TBS emphasised that this depended on patient characteristics, with recovery time longer for older adults. Once healed, as the quotation below highlights, TBS encouraged patients to walk short distances using a home-made walking stick until they were able to walk unassisted:

*“I gave them the local stick and instruct them to walk slowly and do not stand hard on your feet because the bone might move[…] So, I will allow them to walk seven to eight meters from here or ten to fifteen meters while using the stick to assist them. That is how I do it [walking] back and forth [..]So, this is how we do it slowly until it reaches to a point that I feel like they can walk without support then they will drop the stick.”* ***(TBS 03, male).***

TBS followed up patients by either visiting their homes, especially in the case of an older adult, or requesting the patient to visit them. Most TBS mentioned that, while they do suggest a fee for their treatment, typically between 300 dalasi to 1000 dalasi (4.1 to 13.6 USD), patients were able to pay what they could afford. This was particularly for patients with low incomes living in rural areas. In such cases, some TBS mentioned not charging any payment for their services, but instead requested the patient offer prayers on their behalf.

*“...in the coast there are people who pay but here [in the rural area] we are all poor... they will come and you treat them and some will say, “we came, ahh but we have no money with us”. We ask them to pray for us instead of telling you to pay.”* ***(TBS 03, male).***

While there were variations in the materials used, the narratives detailed above indicate that TBS followed a consistent method for treating hip fractures. Notably, this involved treating a hip fracture as a dislocation through pulling, massaging, and re-aligning the limb, followed by a long period of immobilisation, and then gradual reintroduction of walking. As we have also highlighted above, a key feature of TBS practice involved gaining patients’ trust and empathising with their socioeconomic concerns.

### Interaction between biomedical and traditional sectors in the treatment of hip fractures

#### TBS perceptions of biomedical care.

When asked about their views on the use of biomedical care to treat bone-related injuries, TBS acknowledged some key advantages. One advantage of hospital care identified by TBS was the provision of pain relief medication. TBS also noted that biomedical healthcare professionals have a good understanding of the human body, and this enabled them to treat illnesses beyond a TBS’s capacity.

*You know...we [TBS] don’t know how the blood flows and how you should tie someone [apply bandages to a fracture], because many people [TBS] used to fail on that side. We need such kind of training and also on the side of the bones” (* ***TBS 010, male)*.**

However, other TBS believed that the biomedical treatment of hip fractures led to swelling and pressure sores. They reported observing these issues in patients who had sought their care following unsatisfactory treatment of a hip fracture in a hospital. TBS also explained that patients preferred seeking care from them as the cost was lower, they were closer to home to enable support from family members, and because they thought that healing time would be shorter:

*“I can say here [TBS] is cheaper than the hospital because going to the hospital you have to pay for transportation, the place to stay when you don’t have relatives around the hospital, the food you are going to buy […] So, it is easier and cheaper here.” (* ***TBS 03, male).***

Overall, TBS felt that satisfaction with their treatment of fractures had established trust in communities, over generations, contributing towards patients’ preference for seeking their care. As the TBS describes below, patients did not have the same level of trust in hospitals:

*“Some people knew us from the village and established that trust with us. They don’t have that trust in hospital services like they do with the traditional bone setters.” (* ***TBS 02, male).***

According to the healthcare professionals interviewed, there was the perception in communities that the clinical treatment of fractures inevitably led to amputation and permanent disability and, as the healthcare professional explains below, such perceptions have created mistrust in biomedical care:

*“There is a belief that if you treat a hip fracture at the hospital, you will not walk again... it needs to change their perception in the treatment of hip fracture locally to conventional methods”* ***(Male -Medical Officer).***

#### Healthcare professionals’ perceptions of TBS and their practices.

Most healthcare professionals explained that they had limited knowledge of TBS practices in treating fractures and that their perceptions were shaped by their first-hand experiences of treating patients who had initially received care by a TBS. Healthcare professionals described attending to patients with severe complications that they attributed to poor fracture care. Complications included necrosis, swelling, pressure sores, embolism, and misalignment of bones. One healthcare professional explained that patients seeking care from TBS led to delays in hospital attendance which contributed to the risk of amputation. As outlined, perceptions of permanent disability following clinical treatment of hip fractures was seen to lead to community mistrust in biomedical care.

*“...while working at the hospital, there are a lot of cases, and complications that will come and most of them would have visited one or two bone setters for their condition and then they would end up at the hospital. Sometimes for some people, it is very late, and some people will lose their legs and limbs.”* ***(Male-Administrator).***

Given these concerns, some healthcare professionals suggested that TBS should not be permitted to practice in The Gambia:

*“Let them be banned because they have caused a lot of problems and they will continue to do so. Because they are doing the work blindly and will make the patient believe that they are doing their work very well. People will realize that in the years or months they are still in pain and things are not getting better”* ***(Male-Resident Doctor).***

While others also voiced concerns about the harmful consequences of fracture care by TBS, they acknowledged that TBS were an important part of hip fracture management given current challenges in biomedical care in The Gambia:

*“...my opinion about them is they are a critical component of fracture care in our setting here, in this part of the world.... More so in a country where you have only two or three orthopaedic surgeons...having to deal with a population of 2 million. I would be quick to say that if there were not traditional bone healers, we would not be able to deal with the number of fractures...”* ***(Male-Orthopaedic surgeon).***

Healthcare professionals described the various health system challenges they encountered including shortages of bed space and operating theatres, unavailability of implants and pain medication, and the shortage of orthopaedic care staff. Some healthcare professionals thus felt it difficult to completely discount the role of TBS in hip fracture management in The Gambia. This view was more common amongst healthcare professionals who had lived in a village themselves and had family members treated by TBS. Given these considerations, they acknowledged that TBS are often the first point of contact for patients seeking care for hip fractures.

#### Existing informal interactions between TBS and the health system.

Both TBS and healthcare professionals indicated that there remains no formal integration of the two sectors for fracture treatment, including for hip fractures. However, the TBS disclosed that they sometimes directed patients to hospitals, particularly where injuries were considered severe. For instance, some TBS reported referring patients to a hospital in the case of an open fracture (where there is an open wound) or for other non-bone related ailments (including co-morbidities). Moreover, a few TBS felt that hip fractures were serious injuries that warranted referral to a hospital, especially for older adults.

*“[We] just send them to the hospital to fix their hip, especially for older people you know dealing with them is not easy so I just send them to the hospital”* ***(TBS 08, male).***

As described earlier, some TBS referred patients to hospitals for x-rays to identify a fracture, while others mentioned referring patients to hospital if they suspected witchcraft was involved in causing the injury.

In some cases, TBS explained that they were requested by family members to visit the hospital and provide treatment. However, healthcare professionals emphasised that TBS treatment in a hospital setting would not be allowed:

*“They [the patient and family] told us [healthcare professional] not touch the fracture and they wanted the traditional bone setter to come to the hospital to treat the fracture. We told them the TBS cannot come here... we are going to take care of him*.” ***(Male-Resident Doctor).***

Importantly, all participating healthcare professionals stated that they would not refer a patient to a TBS for treatment. Referrals between biomedical and traditional care sectors in The Gambia therefore seemed to be unidirectional.

### Recommendations from TBS and biomedical healthcare professionals

Though there is no formal integration of biomedical and traditional sectors, most TBS were open to collaborating with the biomedical healthcare professionals to manage hip fractures. Indeed, many saw the advantages of such a partnership and recommended establishing a formal collaboration between the two sectors. As one TBS described, they felt that such a collaboration would allow them to access materials they lacked such as walking sticks, bandages, and pain medication:

*“I would be happy to see both traditional bone setters and healthcare professionals work together when a fracture patient is being seen by both parties. I want to see that when a patient is seen by a traditional bone setter and needs pain killers, the hospital should give those medications to the local healer to give to their patient when they pull the fracture”* ***(TBS 015, male).***

However, other TBS felt that such an agreement would be ineffective if the government did not recognise them as “legitimate” healthcare professionals. For this reason, some TBS called for the government to support their practices by offering training, establishing guidelines, and providing funds which would enable them to have their own clinics:

*“...we will need to have the attention of the government by supporting us by giving us money and having rules for our treatment.”* ***(TBS 06, male).***

While TBS generally expressed an openness to collaborate with the biomedical health sector to provide hip fracture care, healthcare professionals remained ambivalent about forming a partnership with TBS, given the complications that they attributed to TBS treatment. This view was particularly prevalent amongst the younger healthcare professionals interviewed who felt that TBS were taking their jobs – jobs which they had worked hard to secure – while discouraging the community from seeking formal health care.

*“We have seen the outcomes, most of the complications of the fractures are osteomyelitis, non-union, and mal-union. We have seen all of these people come to the hospital after the traditional bonesetters are done with them...”* ***(Male-Medical Officer).***

However, other healthcare professionals recommended that TBS should receive training in the appropriate identification and management of fractures, in general, and in infection control and the treatment of open wounds. Healthcare professionals explained that an essential part of the training should be for TBS to recognise when to refer complex cases to a hospital:

*“There is already trust built with them and locally in our context... so they should be able to know their limitations and build that rapport with patients and tell them whenever they think they are not able to handle [cases], they should be able to refer the patient.”* ***(Female-General Nurse).***

Establishing such a referral network, some healthcare professionals argued, would prevent complications and delayed presentation of patients to hospitals. Healthcare professionals emphasised the importance of instituting regulatory mechanisms within TBS practices with appropriate training and certification. Some suggested that these initiatives might be facilitated by the Ministry of Health, who could provide overall regulation and legislation of TBS practice through their dedicated ‘traditional medicine’ branch. They noted the success of other initiatives, such as the training of Traditional Birth Attendants (TBA) in the referral of pregnant women in the community to health facilities for delivery:

*“One of the strategies I would like to recommend is for the Ministry of Health to come up with an initiative like that of the community birth companions (TBAs)... TBAs accompany a woman in labour to the facility they (TBAs) are given D500, (6.8 USD) that is a very good strategy. First, they were reoriented on the importance of institutional delivery and a package was allocated to encourage them to refer these patients, and even come with the patients to the facility. I think we can do a similar thing with the bonesetters.”* ***(Male –Pre-operation Nurse).***

Nevertheless, many healthcare professionals felt that the Ministry of Health should prioritise improving health system capacity to overcome existing barriers to orthopaedic care, such as limited orthopaedic staff. They suggested that such changes could be made alongside the implementation of TBS training initiatives. Healthcare professionals also stressed the importance of community-based initiatives to foster confidence in the effectiveness of hospital treatment for hip fracture.

## Discussion

This study provides an account of TBS practices and processes in the treatment of hip fractures in The Gambia. In doing so, we have highlighted the continuation of TBS practices in the treatment of hip fractures in The Gambia, despite growing biomedical care provision [32]. During the interviews, TBS described the different ways they acquired bone setting knowledge – most were taught by a family member (usually their father), yet a few mentioned that their knowledge was a “gift from God”. That said, we did not observe any differences in bone setting practice based on how TBS acquired their knowledge. Other studies, such as that of Hancock et al. [18], have also noted the acquisition of bone setting skills through “spiritual guidance”, aside from family inheritance. The absence of formal training and licensing of TBS in many parts of Africa has underscored calls to develop TBS training and educational initiatives to reduce the incidence of complications [6,18,19]. Though emphasised as strictly patriarchal by many, some TBS mentioned training their daughters in bone setting – this variation has also been reported in work in Nigeria [33]. TBS described being acutely aware of the difficult socioeconomic circumstances of their patients and, in turn, allowed patients to pay what they could afford. Such a practice enabled patients to overcome financial barriers to accessing hip fracture care in biomedical institutions. As our recent study has confirmed, affordability was one of the main motivators for patients seeking TBS care for hip fractures [25]. This practice is not unique to The Gambia, though, as work by Hancock et al. [18] in Chad noted how TBS or “Jabari” reduced their prices for low-income patients. As majority of TBS did not charge a standard fee, their income from exclusively practicing bone setting was insufficient and the TBS interviewed had a range of primary occupations. This, in turn, enabled TBS to practice in a range of locations and they remained open to treating patients, especially older adults, in their homes. This flexibility can allow patients to access care for a hip fracture when biomedical care may be financially and/or geographically inaccessible. As our work in The Gambia has shown, patients’ and caregivers’ decisions to seek care from a TBS was strongly influenced by convenience [25].

Most TBS, including those using radiographs, diagnosed hip fractures as hip dislocations. Treatment thus involved pulling, massaging, and kneading the limb back into the hip socket. Research conducted in other African settings, including Nigeria, Ghana, and Tanzania, outline similar methods used by TBS to treat fractures [23,24,33,34] following the steps of pulling, bandaging, and splinting. There are, however, differences in the materials used for splinting, the herbal concoctions applied, and analgesics administered for pain [21,23,24]. For instance, in their work in Nigeria, Omololu et al. [24] indicate the use of cardboard and plywood for splinting and the administering of alcohol as an analgesic. This was not reported in our study, likely due to The Gambia being a Muslim-majority country with minimal alcohol consumption. Moreover, though several studies have previously documented TBS use of X-rays in fracture identification, in this study, we found that X-rays also served to prove the efficacy of TBS treatment [21,33], presumably through evidence of callus formation which would be expected to develop whatever the treatment provided. [21,23,24,33–35].

The “spiritual role” of TBS in treating “satanic causes” of fractures varies across studies, as it did among the TBS we interviewed [33,34,36]. While some TBS described reciting incantations from the Quran, others mentioned they would not treat patients if witchcraft was associated with the cause of injury – a finding that is consistent with what is reported in current literature [33,34,36]. Following treatment of the fracture, TBS advised on the strict immobilisation of the limb which could last from two weeks to a few months. Once they deemed a fracture healed, TBS encouraged patients to walk short distances using a home-made walking stick. Previous studies in Nigeria and Tanzania have also described protracted immobilisation of a fracture though, in Aires et al.’s study in Ghana [23], patients were advised to fully use and carry weight on the body part after three to four weeks. This guidance differs notably from the AO Alliance’s clinical guidelines for fracture care, which advocate for full weight-bearing the day after surgery [5]. Clinical research has shown that early mobilisation helps reduce the risk of complications such as venous thromboembolism, pressure sores, acute sarcopenia (muscle loss and weakness), and pneumonia, while also supporting recovery [37]. Given this, we are in the process of carrying out further research to describe treatment outcomes for hip fractures provided by both traditional bone setters and biomedical care. This includes examining patients’ expectations, perceived treatment effectiveness, satisfaction with care, and the barriers and facilitators that influence outcomes.

TBS and healthcare professionals had varying perceptions and views of the respective care sectors. For instance, TBS did acknowledge that one key advantage of the biomedical treatment of hip fractures was the provision of pain relief. However, they noted that high costs of biomedical care, poor accessibility of healthcare facilities, and established community trust contributed towards peoples’ satisfaction with their treatment. Healthcare professionals, on the other hand, described concerns of and experiences with managing complications associated with TBS treatment of hip fractures. This, and the view that TBS were taking their jobs, led some healthcare professionals (particularly younger professionals) to feel that TBS should be banned from practicing in The Gambia. Work by et al. [38] has shown how power dynamics between healthcare professionals and traditional healers can be barriers to integration. In studies conducted in Liberia [39] and Kenya [40], for instance, traditional healers felt that biomedical healthcare professionals had no desire to collaborate due to their traditional practices being viewed as ‘inferior’ and the belief they were less competent. Other work from Ghana [41] suggested that biomedical healthcare professionals’ unwillingness to collaborate stemmed from their scepticism of how traditional healers acquired their knowledge. Nevertheless, in this study, some healthcare professionals acknowledged the health system-related challenges (such as shortages of orthopaedic staff, bed space, and operating theatres) they experienced and, in this way, felt it difficult to discount the role of TBS in hip fracture care provision in The Gambia.

The interviews with both TBS and healthcare professionals identified an absence of formal collaboration or interaction between the two sectors in the treatment of hip fractures in The Gambia. This situation is similar in other African countries, as work by Aries et al. [23] on patients’ experiences of bonesetters in Ghana has shown. That said, in The Gambia, some TBS directed patients to healthcare facilities, mainly to undergo diagnostic radiography, receive pain relief medication, and treatment of co-morbidities. This was not part of a formal referral system and seems to be strictly unidirectional – while TBS described referring certain patients to receive hospital care, biomedical professionals did not report referring patients to TBS. Nevertheless, these findings demonstrate the overlap in practices between biomedical and traditional care sectors in treatment of hip fractures in The Gambia. This overlap has been similarly reported in research conducted in other contexts, such as in Uganda, the Philippines, and Nepal, and for different health conditions, including hypertension and mental illness [13–15]. This evidence echoes recent debates within medical anthropology around viewing “medical pluralism” as a concept which enables researchers to highlight the interactions and overlaps between different therapeutic traditions, rather than examining such traditions as separate and distinct [8,11]. As Krause et al. [8] and others [10] suggest, this conceptual shift can allow researchers to understand how boundaries between traditional and biomedical care are constantly reconfigured in response to interactions between the sectors. These interactions are evident when patients seeking care from these therapeutic traditions at various stages of their treatment journey, as our recent work from The Gambia [25] has highlighted. However, as we have shown in this study, they also occur through formal or informal collaborative activities between biomedical and traditional healthcare professionals.

Most TBS were open to collaborating with the biomedical health sector, though some felt that such partnerships would be ineffective if they were not “legitimised” by the government. On the other hand, many healthcare professionals expressed some hesitancy around forming such collaborations and recommended the need to train TBS in infection control, fracture identification and management, and in the referral of complex cases to hospitals. Other work from Nigeria [13] and Ghana [20] has similarly noted the importance of TBS training initiatives, regulatory mechanisms of TBS practices, and legislation to establish successful collaboration and referral pathways between biomedical and traditional care sectors. The recent 2021–2030 National Health Policy noted significant health system challenges in The Gambia such as, high out-of-pocket health expenditure and insufficient human resources [22]. As noted above, our work identified that less than one orthopaedic surgeon is currently available for each 100,000 adults in The Gambia, with only around 7% of current inpatient care dedicated to orthopaedics. Given this, healthcare professionals emphasised the futility of collaborative initiatives if existing barriers to orthopaedic care are not addressed.

## Recommendations for research, policy, and practice

Considering our analysis and the key objectives from the WHO’s newly published Traditional Medicine Strategy (2025–2035), Table 3 outlines recommendations that could be implemented in the short-, medium-, and longer-term to support collaboration between traditional and biomedical sectors on the provision of hip fracture care in The Gambia [16]. These recommendations were derived from suggestions made by healthcare professionals and TBS during the interviews, framed within the WHO Traditional Medicine Strategy objectives. As described above, the Strategy outlines four strategic objectives including the need to strengthen the evidence base around the safety and effectiveness of biomedical, traditional, and complimentary care, establishing regulatory mechanisms, integrating traditional and complimentary medicine into health systems, and supporting the empowerment of communities to make informed care decisions about their treatment [16]. Interviews with healthcare professional also provided valuable information on the health system context in The Gambia – the nature of orthopaedic care, policy environment, and current health system gaps. These insights were key to understanding the implementation of such recommendations within the context of The Gambia. Interviews with both TBS and healthcare professionals provided insights into community perceptions of traditional and biomedical care. Finally, through joint discussions, we drew on our own expertise as clinicians, social scientists, and health systems and policy researchers to formulate the recommendations in Table 3.

**Table 3:** Recommendations for the collaboration between traditional and biomedical sectors on the provision of hip fracture care in The Gambia.

Recommendation	Period of implementation		
**Short-term**	**Medium-term**	**Long-term**
Development of training and educational programmes for TBS in fracture care (treatment of open fractures, infection control, bandaging and splinting, general anatomy) with appropriate certification			
Develop evidence-based guidelines that specify cross-sectoral referrals between biomedical and TBS care. This includes guidance for TBS on the referral of complex fractures			
Political commitment and support from the Ministry of Health on integrating biomedical and traditional care into secondary- and tertiary-levels of the health system			
Workshops to facilitate intersectoral dialogue and communication between health system actors (policymakers, healthcare professionals, facility managers, researchers) and TBS			
Establish regulatory mechanism for TBS practices, including establishing quality control measures, licensing requirements and a TBS registry			
Co-designing of collaborative initiatives which involve community members, TBS, and health system actors (including policymakers, healthcare professionals, facility managers, researchers)			
Remove geographical barriers to accessing biomedical care by improving road conditions and networks for better transportation access (including ambulance access)			
Implement community-based educational campaigns for the biomedical treatment of hip fractures			
Continued research to strengthen the evidence base for integration of traditional and biomedical sectors of care			

Short-term recommendations are those that are immediately actionable within the healthcare system. As such, these actions should be prioritised in the short term as they can be feasibly integrated within existing systems and procedures. Medium‑ and long‑term recommendations are also feasible but require additional engagement and development. Medium-term recommendations will require collaboration between key stakeholders, including TBS, policymakers, and health system actors (healthcare professionals, facility mangers, researchers) to be feasibly implemented within The Gambia’s health system. Finally, longer-term recommendations will require extensive collaboration between these stakeholders. Some initiatives can (and should) be implemented in the short-, medium-, and longer-term, such as the need for political commitment and support for the collaboration between the biomedical and traditional sectors for hip fracture care.

The WHO’s Strategy calls for the reorientation of health services to integrate traditional care into the formal health system [16]. They propose that, as the foundation of universal health coverage, primary healthcare would be the “natural entry point” for this integration. However, as noted in the National Health Policy, the primary healthcare system in The Gambia has only about 40% coverage across the country. Moreover, orthopaedic care is currently only provided at three central hospitals [27]. Hence, we propose the potential integration of TBS services into secondary- and tertiary-levels of the health system in The Gambia. While the WHO Strategy advocates for the integration of traditional care at the primary care-level care, this may not always be feasible or appropriate in certain contexts and for given conditions, such as fractures [16].

There are several existing initiatives that could serve as models for implementing these recommendations. The African Orthopaedic Alliance, a non-governmental organisation, has previously implemented and funded a TBS training programme in Ghana [42]. The programme was developed by a multidisciplinary team of experts (orthopaedic surgeons, and public health experts, physiotherapists) and included interactive lectures and practical exercises in the local language [42]. The effectiveness of the programme suggests that a similar training programme can be implemented in other contexts such as The Gambia. Moreover, during the interviews, healthcare professionals noted the need for government support, through the Ministrywo of Health, in providing overall oversight and regulation of TBS practice. They pointed out the success of the Traditional Birth Attendant initiative, proposing that a similar model could be used for TBS in The Gambia. Overall, as also outlined within the WHO Strategy, we note the importance of continued research to strengthen the evidence base for integrating traditional and biomedical sectors for fracture care. This includes research which analyses community perceptions of TBS practices, in The Gambia and globally.

## Strengths and limitations

In this study, we have described the intricacies of TBS practices and the context in which they treat hip fractures in The Gambia. Interviews with both TBS and healthcare professionals enabled us to capture the differing views they held about traditional and biomedical care sectors, and their varying recommendations for collaboration. The inclusion of TBS from both urban and rural areas of the country further highlighted the influence their geographical location had on their practice (such as treating rural patients in their homes). Moreover, we used information power to ensure our final sample size was sufficient to address the study aims [25].

While most TBS appeared to be willing to share information about their procedures and materials, some were hesitant to describe certain details of their practices such as the materials they used and/or how they acquired their bone setting knowledge. They explained this was related to their wish to preserve bone setting knowledge and therefore only communicate such information to selected individuals. We also acknowledge the possibility of social desirability bias in which some TBS may have felt the need to present themselves in a favourable light to researchers working within biomedical institutions [43]. Given that several TBS thought that biomedical healthcare professionals were seen as having a higher status within the healthcare system than TBS, TBS participants may have emphasised successful treatment outcomes or underreported complications. Moreover, we recognise that recruitment of TBS was limited to two locations in The Gambia and that it is possible that TBS from different lineages may have different bone setting practices. Nevertheless, we are confident that we have included rich descriptions of the treatment of hip fractures by TBS in The Gambia. The depth of material collected was also facilitated through our iterative approach to data collection and analysis, in which we refined data collection tools to explore early insights as they were identified in analysis. This ensured that data collected explored and sufficiently explained unexpected findings we did not initially consider at the beginning of data collection. Data analysis was conducted by a research team (AT, SD, and KL) who have in-depth understanding of the study context and the methodologies used. Collaborative meetings between the research team enabled ongoing discussion and refinement of the findings and contributed towards rigorous analysis by enabling us to incorporate the views and experiences of researchers working in different contexts and from different disciplinary backgrounds.

## Conclusion

A growing ageing population in Africa points to the need to reorient health systems to provide care for non-communicable conditions that affect those in mid-life and older age. In countries such as The Gambia, although the impact of hip fracture on individuals is considerable, research has identified hip fracture care provision as an area where supply is not meeting need. Our research highlights the key role TBS play in hip fracture care provision in The Gambia. We have also described the interactions between both TBS and biomedical care in the treatment of hip fractures. However, despite international calls to integrate traditional and biomedical sectors of care, we identified the unidirectional referral of hip fracture patients from TBS to biomedical care. While most TBS were open to collaborating with the biomedical health sector to manage hip fractures, some biomedical healthcare professionals noted their desire for regulation of TBS practices and training initiatives. These insights have enabled us to propose short-, medium- and long-term recommendations to promote the integration of traditional and biomedical sectors in the treatment of hip fractures in The Gambia.

## Supporting information

S1 Text Topic guide for interviews with TBS. (DOCX)

S2 Text Observations checklists for observations of TBS practice. (DOCX)

S3 Text Topic guide for interviews with healthcare professionals. (DOC)
