# Supplementary material for: Traditional bone setter practices and the interaction with biomedical care in the treatment of hip fractures in The Gambia: A qualitative study
Source: PLOS Glob Public Health. 2026 Jul 14;6(7):e0006582. doi: 10.1371/journal.pgph.0006582 (PMC13367902; doi:10.1371/journal.pgph.0006582)
Supplement: S1 Text — (DOCX) [file pgph.0006582.s001.docx]

**Fractures in Sub-Saharan Africa – The Fractures E3 Study**

**A study of hip fracture care**

**TOPIC GUIDE TRADITIONAL BONE SETTERS**

Interviews will focus on the topics below and will be informed by observational fieldwork.

**Aims and objectives**

- To find out more about the treatment that is available for people who have broken their hip
- This project will provide us with information that can be used to improve the care that is available to people who have broken their hip.

**Introduction**

- Introduce self
- Introduce the study: who is it for, what is it about
- Key points - Length of interview, voluntary nature of participation and right to withdraw, recording of the interview
- Confidentiality and how findings will be reported - No names, published in reports and academic publications, short quotes from them in write-up
- Any questions that they have
- Take consent – signed consent, thumb print (with witness), verbal (audio recorded)

**Part 1: Find out a bit about you**

- Job name
- Years spent working as a bone setter
- Around how many hip fracture patients they see each month
- Why they decided to be bone setter
- Do you have another occupation different from this?
- If yes, what is your other job?
- Why do you have another job?
- Age
- Gender
- Tribe
- Why do you choose this location?

**Part 2: Understanding treatment**

- Describe what happens in clinic/ service
- Describe what they do in treating people who have broken their hip
- How identify broken hip, e.g. use of x-rays
- Treatment, e.g. splinting, re-setting
  - What is splinting made of
  - How is this done
- Anything else done to help healing, e.g. herbal creams

What do they do when someone is in pain?

- Rehabilitation, e.g. exercises, bed rest, mobility aids
- Follow-up visits from clients
- Managing complications, e.g. refer to more experienced TBS or hospital
- Do they keep records of people they have treated?
- Does anyone help them when treating patients, e.g. other members of the family
- How do these people help?
- Payment

**Part 3: Patients**

- Types of patients that visit
- Why they think patients visit traditional bone setters

**Part 4: Relationship with healthcare services**

- Bone setters vs healthcare services – advantages/ disadvantages
- What do they think about hospital services?
- What do you think hospital services think of traditional bone setters?
- Communication with healthcare professionals
- Referrals to healthcare services
- When?
- Why?

**Part 5: Training and knowledge**

- How they learnt to be bone setter, e.g. training, who delivered this training?
- How is this knowledge transferred from one person to another
- Do they train others?
- What does training other bone setters involve?
- How do you regulate practices

**Part 4: Close**

- What could be done to improve care for people who have broken their hip
- Any other issues to discuss
- Questions about the project
- Thank them
- Reaffirm confidentiality
