# Supplementary material for: Traditional bone setter practices and the interaction with biomedical care in the treatment of hip fractures in The Gambia: A qualitative study
Source: PLOS Glob Public Health. 2026 Jul 14;6(7):e0006582. doi: 10.1371/journal.pgph.0006582 (PMC13367902; doi:10.1371/journal.pgph.0006582)
Supplement: S3 Text — (DOC) [file pgph.0006582.s003.doc]

SCC/Protocol No:__________________

For office use only: to be completed by research staff

| Site |  |  |  | Participant ID |  |  |  |
| --- | --- | --- | --- | --- | --- | --- | --- |

**Fractures in Sub-Saharan Africa – The Fractures E3 Study**

**A study of hip fracture care**

**OBSERVATION SCHEDULE TRADITIONAL BONE SETTERS**

| **Details of observation session:**     | **Location:** |  | | --- | --- | | **Time and date:** |  | | **Observation number:** |  | | **Length of time spent observing:** |  |   **Topics to explore:**   - Description bone setters, numbers - Description of patients and carers - Description of setting including layout of working area - Activities taking place, including treatments - Interactions between bone setters, e.g. types of bone setters, hierarchies, distribution of work - Interactions between patients and bone setters |  |
| --- | --- | --- | --- | --- | --- | --- | --- | --- | --- |
